# Comparisons between two biochemical markers in evaluating periodontal disease severity: a cross-sectional study

**DOI:** 10.1186/1472-6831-14-107

**Published:** 2014-08-30

**Authors:** Sakornrat Khongkhunthian, Prachya Kongtawelert, Siriwan Ongchai, Peraphan Pothacharoen, Thanapat Sastraruji, Dhirawat Jotikasthira, Suttichai Krisanaprakornkit

**Affiliations:** 1 Department of Restorative Dentistry and Periodontology, Faculty of Dentistry, Chiang Mai 50200, Thailand; 2 Department of Biochemistry, Thailand Excellence Center for Tissue Engineering and Stem Cells and Center of Excellence for Innovation in Chemistry, Faculty of Medicine, Chiang Mai 50200, Thailand; 3 Department of Oral Biology and Diagnostic Sciences, Center of Excellence in Oral and Maxillofacial Biology, Faculty of Dentistry, Chiang Mai 50200, Thailand; 4 Department of Orthodontics and Pediatric Dentistry, Faculty of Dentistry, Chiang Mai University, Chiang Mai 50200, Thailand

**Keywords:** Alkaline phosphatase, Chondroitin sulfate, Chronic periodontitis, Gingival crevicular fluid

## Abstract

**Background:**

The purpose of this study was to compare two biochemical markers, which have been previously used to determine the degrees of alveolar bone destruction, in evaluating periodontal disease severity.

**Methods:**

The WF6 epitope of chondroitin sulfate (CS) and the alkaline phosphatase (ALP) levels were determined in gingival crevicular fluid (GCF) samples collected from patients with various degrees of disease severity, including ten patients with gingivitis (50 gingivitis sites) and 33 patients with chronic periodontitis (including gingivitis, slight, moderate, and severe periodontitis sites; *n* = 50 each), as well as from ten healthy volunteers (50 healthy sites) by Periopaper strips. The levels of CS and ALP were measured by an ELISA and a fluorometric assay, respectively.

**Results:**

The results demonstrated low levels of CS and ALP in non-destructive and slightly destructive periodontitis sites, whereas significantly high levels of these two biomolecules were shown in moderately and severely destructive sites (*p* < 0.05). Although a significant difference in CS levels was found between moderate and severe periodontitis sites, no difference in ALP levels was found. Stronger correlations were found between CS levels and periodontal parameters, including probing depth, loss of clinical attachment levels, gingival index and plaque index, than between ALP levels and these parameters.

**Conclusions:**

It is suggested that the CS level is a better diagnostic marker than the ALP level for evaluating distinct severity of chronic periodontitis.

## Background

At present, diagnosis of periodontal diseases and evaluation of their severity are based on conventional clinical parameters and radiographic findings. However, these methods cannot rapidly detect early periodontal tissue losses and are, therefore, insufficient to determine the degrees of disease severity. Therefore, different types of adjunctive tools are introduced into clinical practice and provide more validity for correct diagnosis and proper treatment planning [1]. Up to the present, several biomarkers have been used for diagnosis and assessment of periodontal disease status as well as prognostic prediction of periodontal disease progression because of their sensitivity and specificity. A number of previous studies have recommended gingival crevicular fluid (GCF), a serum exudate, as a source of biomolecules sampling in order to evaluate periodontal disease status [2-4]. GCF constituents are composed of more than 65 components that have been reported as possible biomarkers for periodontal disease progression [5]. These include inflammatory mediators and host-response modifiers [6-8], host-derived enzymes and their inhibitors [9-11] and tissue breakdown products [12-14].

Among host-derived enzymes, positive association between alkaline phosphatase (ALP) and periodontal disease activity has previously been reported [15,16]. This membrane-bound glycoprotein, which functions as a phospho-hydrolytic enzyme, is a calcium- and phosphate-binding protein [17]. ALP is released from neutrophils and, thus, detected in GCF collected from inflamed periodontium as well as from osteoblasts during bone formation [18,19]. With respect to tissue-breakdown products, several studies have investigated the levels of proteoglycans and their constituent glycosaminoglycans (GAGs) in periodontal tissues and GCF from patients with periodontal disease in comparison to those from healthy controls [20,21]. Major GAGs present in periodontal tissues are dermatan sulfate, hyaluronan, chondroitin sulfate (CS) and keratan sulfate. A main source of GAGs in GCF is derived from extracellular matrix degradation of periodontal tissues during the progression of periodontal disease. Consequently, raised levels of GAGs detectable in GCF directly reflect, and are associated with the destruction of periodontal tissues, especially alveolar bone, at the diseased site [22-25]. Furthermore, elevated levels of CS in GCF are apparently associated with four clinical parameters of periodontal status, which serve as gold standards for periodontal inflammation and destruction [14]. In addition to periodontal disease, CS levels can be precisely detected in physiologic tooth movement [26,27] and in patients with pathologic inflammatory disorders, such as degenerative joint diseases [28,29].

Although a number of studies have examined the relationship of each individual biochemical marker to different types and severity of periodontal diseases, there is still no study comparing the efficiency of different markers in evaluating disease severity. The aims of this study were, therefore, to investigate the levels of CS, as determined by our patented monoclonal antibody, raised against the WF6 catabolic epitope of CS [30], and of ALP in GCF obtained from patients with various disease statuses in comparison to healthy controls, and to compare the efficiency of these two biochemical markers in assessing the severity of periodontal diseases.

## Methods

### Participants

Forty-three patients, including 10 patients with gingivitis (5 males and 5 females) and 33 patients with chronic periodontitis (16 males and 17 females), were recruited from the Periodontology clinic, Faculty of Dentistry, Chiang Mai University along with ten healthy volunteers (5 males and 5 females). Written informed consent for participation in this study was obtained from all participants. They were diagnosed according to the classification of periodontal diseases by the American Academy of Periodontology (AAP) 1999 [31]. The periodontal tissues of healthy controls did not exhibit any clinical signs of inflammation or destruction, whereas those of patients with gingivitis showed clinical signs of gingival inflammation and bleeding upon probing without periodontal pocket formation. The clinical features of patients with chronic periodontitis comprised gingival inflammation and bleeding and periodontal pocket formation from alveolar bone loss as evaluated by the radiography. Patients and volunteers with underlying systemic diseases were excluded from this study according to their medical history obtained from an interview with dental practitioners at the Oral Diagnostic Clinic, Faculty of Dentistry, Chiang Mai University. The participants recruited in this study were non-smoking and non-pregnant persons without periodontal treatment or a history of antibiotic or NSAID uses within three months prior to GCF sampling. The proposal was approved by the Human Experimentation Committee, Faculty of Dentistry, Chiang Mai University.

### Site selection

Five healthy sites from each healthy volunteer were selected as the healthy group (H), while five gingivitis sites from each patient with gingivitis were chosen as the gingivitis group (G). For patients with chronic periodontitis a total of 200 sites were randomly selected and divided into four groups according to a clinical attachment loss (CAL). Fifty sites with clinical signs of gingival inflammation and bleeding on probing without attachment loss were identified as gingivitis sites in the periodontitis group (PG), 50 sites with CAL 1–2 mm were identified as slight periodontitis group (PSL), 50 sites with CAL 3–4 mm were identified as moderate periodontitis group (PM), and 50 sites with CAL > 4 mm were identified as severe periodontitis group (PSE).

### Periodontal parameters

Clinical parameters, including probing depth (PD), gingival recession, CAL, plaque index (PI) [32] and gingival index (GI) [33] were recorded. The gingival recession and PD were measured using a PCP-UNC15 probe (Hu-Friedy, Chicago, IL, USA) from a reference point, the cementum-enamel junction, and both data were then combined and reported as CAL. All parameters were examined by one experienced periodontist (S.K.). The intra-examiner calibration was performed with 98% and 96% agreement for PD and CAL, respectively.

### GCF sample collection

One week before GCF collection, all clinical parameters were recorded to avoid blood contamination during GCF sampling. The GCF collecting technique was performed as described previously [14]. The selected site was isolated with cotton rolls and gently air dried with a triple syringe. Periopaper strips (ProFlow™, Amityville, NY, USA) and an analytical instrument (Periotron 8000™, Oralflow Inc., Plainview, NY, USA) were used to collect and measure the GCF volumes ranging from 0.04 to 1.76 μl. All of the GCF samples were individually stored at −80°C for further analyses. To recover biomolecules from Periopaper strips, a 100-μl quantity of phosphate-buffered saline was added and then agitated for 30 min at room temperature. The GCF was recovered from the Periopaper strips as previously described [14] with the recovery rate approximately 98%.

### Competitive inhibition ELISA with WF6 monoclonal antibody (mAb)

To determine CS (WF6 epitope) levels, a quantitative ELISA method was performed using our WF6 mAb as described previously [14]. Briefly, microtiter plates (Maxisorp®, Nunc, Roskilde, Denmark) were coated with 10 μg/ml shark PG-A_1_ fraction (100 μl/well) [34] in coating buffer (20 mM sodium carbonate buffer, pH 9.6) overnight at ambient temperature. The plates were then washed three times with 150 μl/well of Tris-incubation buffer (Tris-IB) [34] and dried. One hundred and fifty μl per well of 1% (w/v) bovine serum albumin (BSA) in Tris-IB was added to all plates and then incubated at 37°C for 60 min. Thereafter, 100 μl/well of the mixture, comprising 10 μl of GCF samples or of standard competitors (Shark PG-A_l_D_l_ fraction; ranging from 39.06 to 10,000 ng/ml) in mAb against the WF6 epitope of CS (patent number WO 2005/118645 A1) at 1:100 dilution, were added in duplicate for 60 min at 37°C. Subsequently, the plates were washed, and the IgM-specific peroxidase-conjugated anti-mouse immunoglobulin (100 μl/well; 1:2,000) was added and incubated at 37°C for 60 min. The plates were washed and the peroxidase substrate (100 μl/well) was added at 37°C for 20 min to allow the color to develop. The reactions were stopped by addition of 50 μl/well of 4 M H_2_SO_4_. The absorbance ratio at 492:690 nm was measured using a Titertek Multiskan® MCC/340 multiplate reader (ICN/Flow Laboratories, Costa Mesa, CA, USA). The minimal detection level of ELISA for CS was 0.019 ng/ml. The CS concentration in each sample was normalized by its GCF volume, as measured by Periotron 8000™ [14].

### Determination of ALP levels

The GCF samples were measured for ALP levels by using an Alkaline Phosphatase Detection kit (Sigma-Aldrich, St. Louis, MO, USA) according to the manufacturer’s instructions. Briefly, a 180 μl-quantity of fluorometric assay buffer was added to the mixture that contained 20 μl of GCF sample solution and 1 μl of 10 mM substrate solution (4-methylumbelliferyl phosphate disodium). Then, the mixtures were read at 360 nm for excitation wavelength and 440 nm for emission wavelength in triplicate using a Fluorometer (Synergy H4 Hybrid Multi-Mode Microplate Reader, Biotek®, Winooski, Vermont, USA). Known concentrations of an ALP control (Sigma-Aldrich) were prepared with the dilutions ranging from 0 to 1000 ng/ml. The concentrations of ALP in the GCF samples were measured and calculated from a standard curve of these known concentrations. The ALP concentration in each sample was normalized by its GCF volume, as measured by Periotron 8000™ [14].

### Statistical analysis

The average age of participants was compared between groups by the paired *t*-test. The differences in clinical parameters between groups were analyzed by One-way ANOVA followed by Tukey’s post hoc test. The Kolmogorov-Smirnov test was used to determine the distribution of CS and ALP levels. The differences in the CS or the ALP levels among different severities of periodontal diseases were determined by the Wilcoxon signed-rank test, and the differences between the two groups of severities were determined by the Mann–Whitney *U*-test. The correlations between CS or ALP levels and clinical parameters were determined by Spearman’s correlation coefficient. The results were considered statistically significant when *p*-values were less than 0.05. Data were analyzed by using the Statistical Package for Social Sciences version 17.0 for Windows (SPSS Inc., Chicago, IL, USA).

## Results

### Demographic data and periodontal parameters

Mean ages of healthy controls, patients with gingivitis, and patients with chronic periodontitis, were 27.80 ± 5.18, 26.5 ± 4.79 and 50.27 ± 9.67 years, respectively. No significant difference between the mean age of healthy controls and that of patients with gingivitis was found, whereas the mean age of patients with chronic periodontitis was significantly higher than that of the healthy controls and of the gingivitis group (*p* < 0.001). All four clinical parameters are illustrated in Table 1. With regard to clinical parameters of periodontal destruction, there were no significant differences in mean PD and CAL values between the healthy (H), gingivitis (G) and gingivitis sites in chronic periodontitis (PG) groups (Table 1). However, in patients with chronic periodontitis, mean PD and CAL values in the slightly (PSL), moderately (PM), and severely (PSE) destructive sites were progressively increased and to a significantly greater degree than those of non-destructive sites (*p* < 0.05) (Table 1). With respect to clinical parameters of periodontal inflammation, it was demonstrated that mean GI and PI scores of the PM and PSE groups were significantly higher than those of the other groups (*p* < 0.05) (Table 1).

**Table 1:** A summary of four periodontal parameters (average ± SD) within all studied groups

**Periodontal parameters**	**H (n = 50)**	**G (n = 50)**	**Chronic periodontitis**			
			**PG (n = 50)**	**PSL (n = 50)**	**PM (n = 50)**	**PSE (n = 50)**
**PD (mm)**	2.60 ± 0.50^a^	3.00^b^	2.66 ± 0.48^ab^	3.90 ± 0.36^c^	5.32 ± 0.47^d^	7.28 ± 1.23^e^
**CAL (mm)**	0.00^a^	0.00^a^	0.00^a^	1.88 ± 0.33^b^	3.86 ± 0.78^c^	7.90 ± 1.45^d^
**GI**	0.00^a^	1.00^b^	1.02 ± 0.14^b^	1.08 ± 0.27^b^	1.54 ± 0.54^c^	2.26 ± 0.60^d^
**PI**	0.66 ± 0.52^ab^	1.36 ± 1.08^c^	0.52 ± 0.61^a^	1.06 ± 0.59^bc^	1.78 ± 0.74^d^	2.42 ± 0.67^e^

Different letters from a to e in each row represent an order of the averages from the lowest to the highest values and indicate statistically significant differences (*p* < 0.05) between the studied groups, [healthy sites **(H)**; gingivitis sites **(G)**; gingivitis sites in chronic periodontitis **(PG)**; slight periodontitis sites **(PSL)**; moderate periodontitis sites **(PM)**; and severe periodontitis sites **(PSE)**], within each of the four periodontal parameters, including probing depth **(PD)**, clinical attachment loss **(CAL)**, gingival index **(GI)**, and plaque index **(PI)**.

### Elevated CS and ALP levels in destructive sites of chronic periodontitis

**Figure 1:**
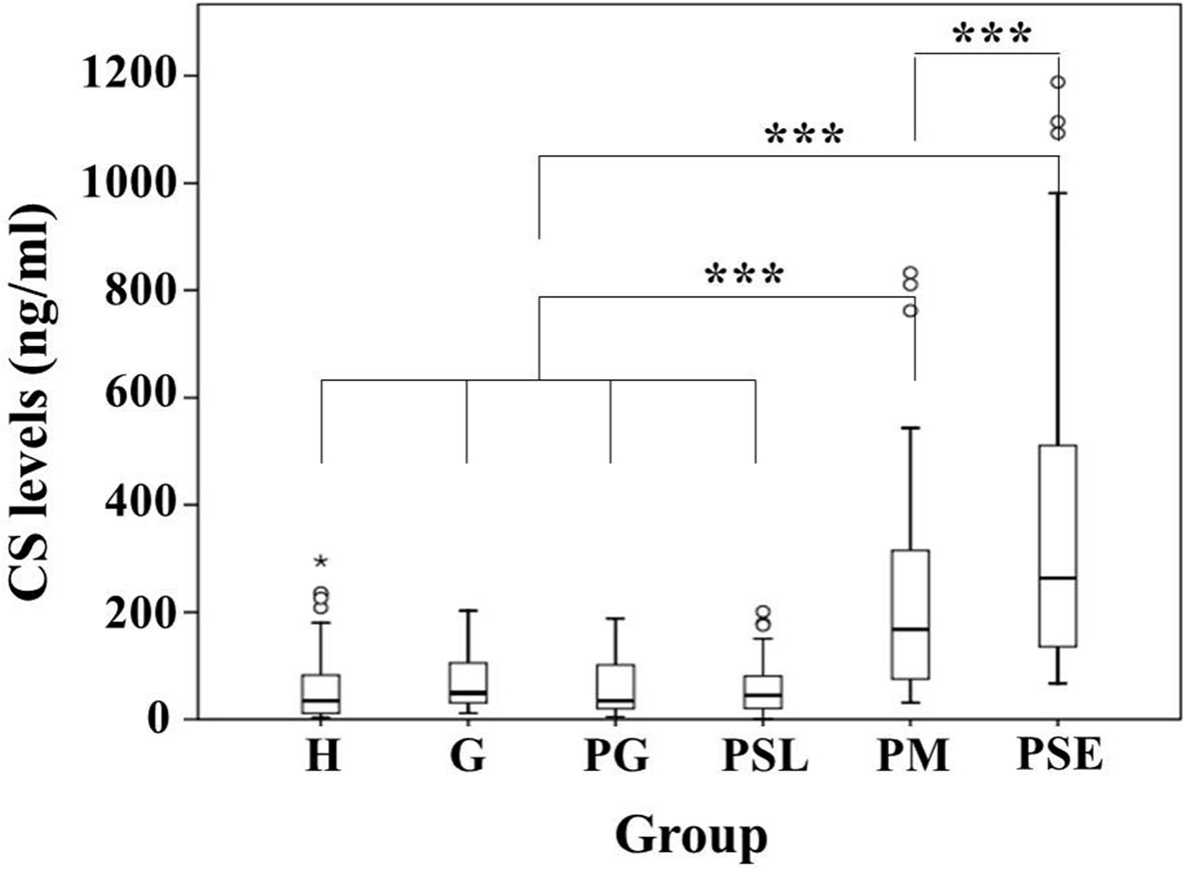
**Raised chondroitin sulfate (CS) levels in gingival crevicular fluid of patients with chronic periodontitis.** The *y*-axis represents the median levels of CS in ng/ml, while the *x*-axis represents various groups of periodontal statuses. **H** = healthy, **G** = gingivitis, **PG** = gingivitis sites in chronic periodontitis, **PSL** = slight chronic periodontitis sites, **PM** = moderate chronic periodontitis sites, **PSE** = severe chronic periodontitis sites. Small open circles and small asterisks are outliers and extremes, respectively. ***p < 0.001.

**Figure 2:**
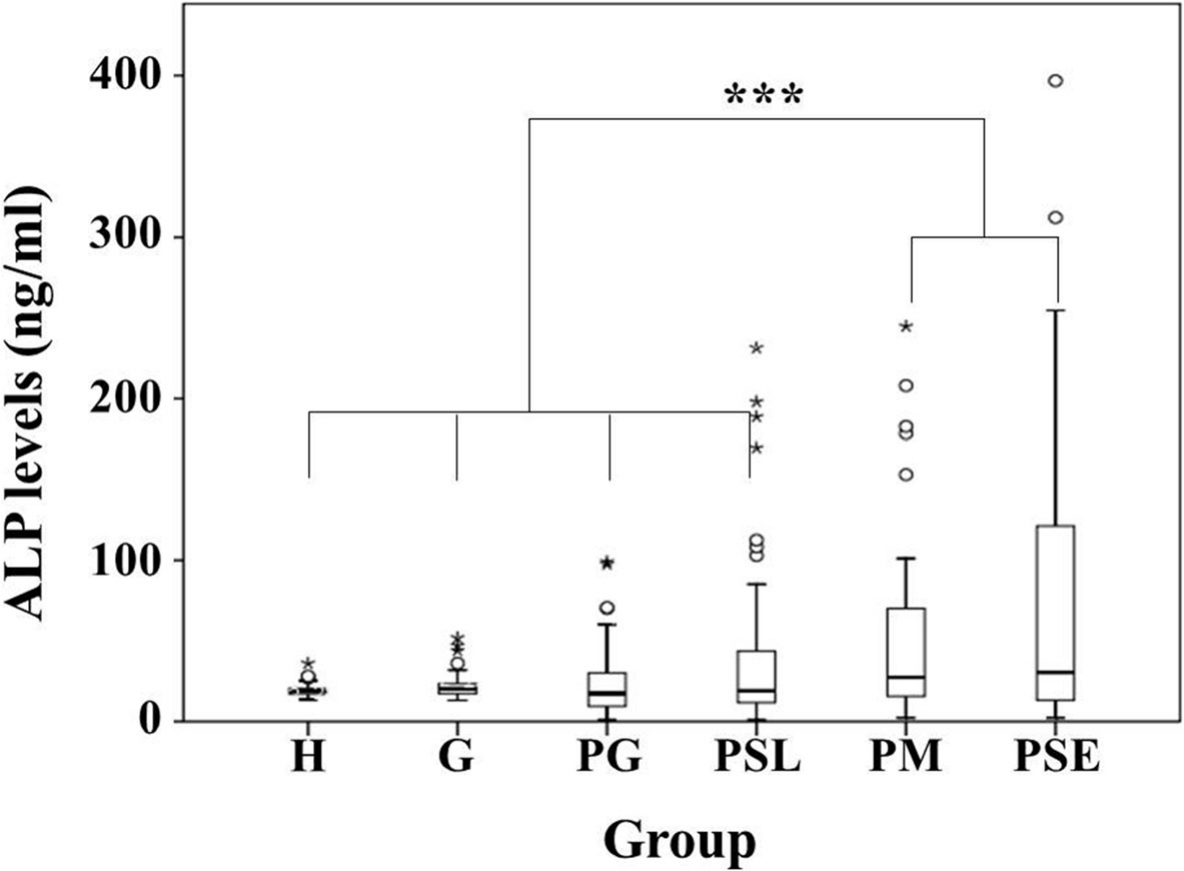
**Elevated alkaline phosphatase (ALP) levels in gingival crevicular fluid of patients with chronic periodontitis.** The *y*-axis represents the median levels of ALP in ng/ml, whereas the *x*-axis represents various groups of periodontal statuses. **H** = healthy, **G** = gingivitis, **PG** = gingivitis sites in chronic periodontitis, **PSL** = slight chronic periodontitis sites, **PM** = moderate chronic periodontitis sites, **PSE** = severe chronic periodontitis sites. Small open circles and small asterisks are outliers and extremes, respectively. ***p < 0.001.

Since the data distributions of CS and ALP levels were not normal, median values of these levels and non-parametric statistical methods were used to determine the differences among different degrees of disease severity. It was found that the median CS levels among the H (28.35 ng/ml), G (40.70 ng/ml), PG (34.80 ng/ml), and PSL groups (45.05 ng/ml) were not significantly different, while those of the PM (168.30 ng/ml) and PSE groups (330.15 ng/ml) were significantly higher than those in the other four groups (*p* < 0.001) (Figure 1). Furthermore, a significant difference in median CS levels between moderately and severely destructive sites (PM vs. PSE) was found (*p* < 0.001) (Figure 1). With respect to ALP levels, no significant differences between the H (19.10 ng/ml), G (21 ng/ml), PG (17.40 ng/ml) and PSL groups (19.10 ng/ml) were observed (Figure 2). In contrast to CS levels, the median ALP levels between moderately and severely destructive sites [PM (27.40 ng/ml) vs. the PSE group (37.05 ng/ml)] were not significantly different, although a significant difference was still observed between non-destructive to slightly destructive sites and moderately to severely destructive sites (*p* < 0.001) (Figure 2).

### Strong correlations observed between CS levels and periodontal parameters

**Figure 3:**
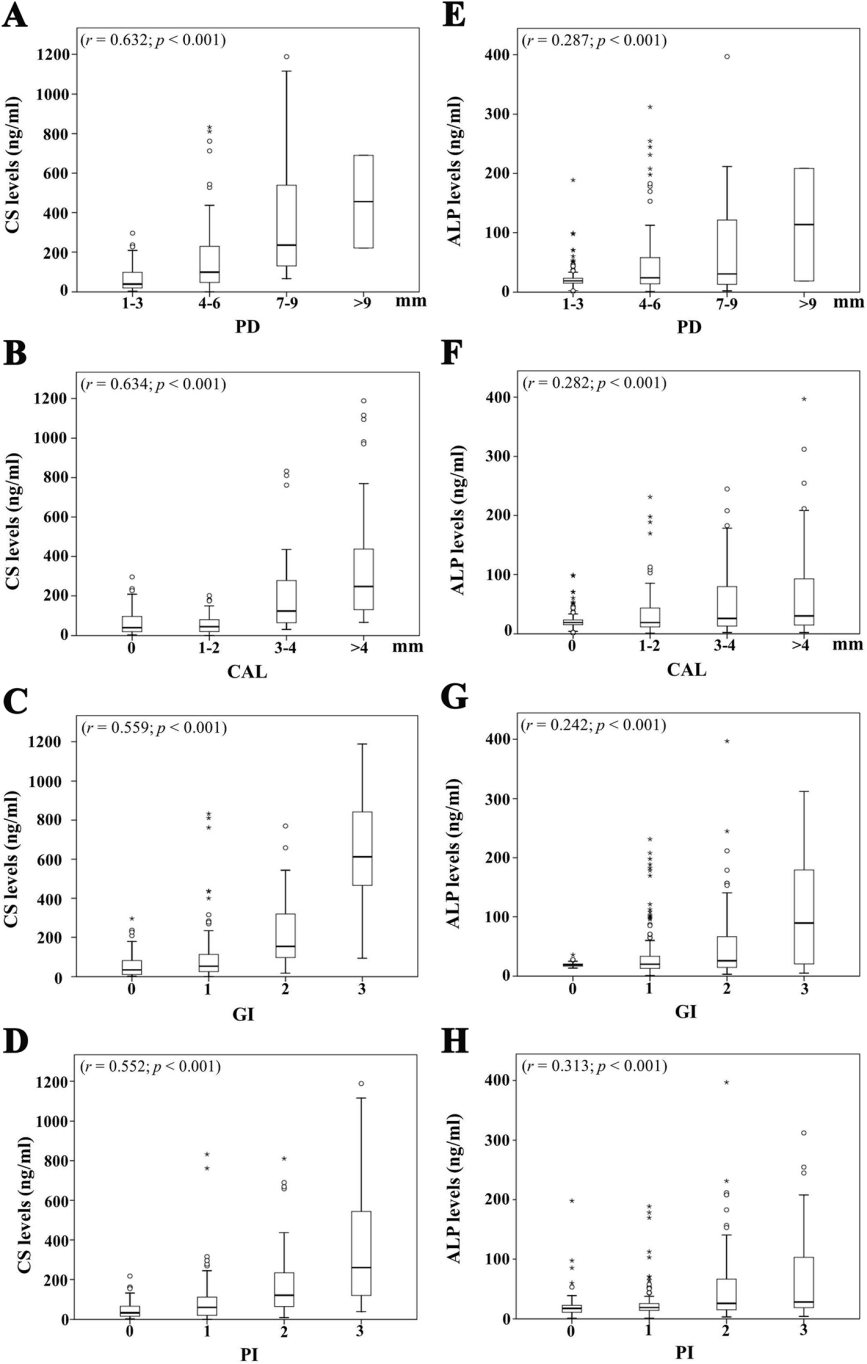
**Significant and positive correlations between the levels of either chondroitin sulfate (CS) or alkaline phosphatase (ALP) and four clinical parameters.** The CS levels in ng/ml **(A, B, C, D)** or the ALP levels in ng/ml **(E, F, G, H)** were associated with four periodontal parameters, including probing depth (PD) in mm **(A and E)**, loss of clinical attachment levels (CAL) in mm **(B and F)**, gingival index (GI; **C** and **G**) and plaque index (PI; **D** and **H**). Note stronger correlations were found between CS levels and all four clinical parameters than between ALP levels and these parameters.

Correlations between CS or ALP levels and four periodontal parameters, including PD, CAL, GI and PI, were determined as shown in Figure 3. It was found that the CS concentrations were significantly correlated with PD and CAL values (*r* = 0.632 and 0.634, respectively, *p* < 0.001) (Figure 3A and B), whereas the ALP levels were weakly correlated with these values (*r* = 0.287 and 0.282, *p* < 0.001) (Figure 3E and F), indicating that the CS levels were associated with the degrees of periodontal tissue destruction more than were the ALP levels. Moreover, the CS concentrations were significantly correlated with GI and PI scores (*r* = 0.559 and 0.552, respectively, *p* < 0.001) (Figure 3C and D), whereas the ALP levels were slightly correlated with these values (*r* = 0.242 and 0.313, *p* < 0.001) (Figure 3G and H), reflecting that the correlation between the CS levels and the degrees of periodontal tissue inflammation is stronger than that between the ALP levels and the degrees of inflammation. In sum, these findings suggest that raised CS levels in GCF represent the degrees of periodontal tissue destruction and inflammation better than do elevated ALP levels.

## Discussion

In this study, it was demonstrated that both CS and ALP levels in GCF collected from patients with different periodontal disease statuses were raised in accordance with the severity of periodontal destruction. Low but detectable CS and ALP levels were observed in the H, G, PG and PSL groups, whereas these levels were significantly elevated in the PM and PSE groups when compared with the non-destructive to slightly destructive periodontitis groups. Interestingly, a significant difference in terms of CS levels between moderate and severe chronic periodontitis sites was demonstrated, whereas no significant difference in ALP levels was found. Furthermore, the levels of both biomolecules were significantly correlated with all of four periodontal parameters, including the degrees of periodontal destruction (PD and CAL) and of inflammation (GI and PI), but stronger correlations between all parameters and CS levels than between those and ALP levels were evident. The reason we chose to study the GCF levels of CS and ALP was because it was previously demonstrated that elevated levels of these two biomolecules were closely associated with alveolar bone destruction in chronic periodontitis [14,35], whereas raised levels of other biomolecules, such as host-derived pro-inflammatory mediators and proteolytic enzymes, can reflect enhanced inflammation and destruction of both soft and hard periodontal tissues. Therefore, we believe that among a number of biomolecules found within GCF, CS and ALP are good candidates for this comparative study to assess the different severities of alveolar bone destruction in chronic periodontitis.

As anticipated, the average age of patients with chronic periodontitis was more than those with gingivitis and healthy controls due to the chronic nature of periodontitis, which is caused by dental plaque accumulation and persistent inflammation of nearby periodontal tissues. Nevertheless, the CS and ALP levels from the gingivitis sites of patients with chronic periodontitis (PG) were not significantly different from those of both patients with gingivitis (G) and of healthy participants (H), although the ages of patients and healthy volunteers in this study were not matched. In contrast to the age difference between the chronic periodontitis and the remaining groups, the gender distribution was not different among groups to avoid bias in the study design. Furthermore, no significant differences in clinical parameters between the H, G and PG groups were found, whereas such parameters in PSL, PM and PSE groups were enhanced according to the severity of periodontitis.

In other previous reports, the studied cohorts were mostly defined as healthy, gingivitis and chronic periodontitis [13,35]. However, in our study, the chronic periodontitis group was further divided into subgroups, including PG, PSL, PM, and PSE, according to the disease severity. We believe that detailed classification of chronic periodontitis according to the severity of alveolar bone destruction will better reflect the potential of biochemical markers to distinguish different disease statuses that can provide useful information and help clinicians in proper treatment planning and periodontal maintenance, while the conventional clinical parameters cannot [36,37].

In some previous studies, the association between ALP and periodontal disease was reported, especially in active diseased sites [16,35]. Significantly higher concentrations of ALP were observed in periodontitis than in healthy and gingivitis sites, and positive correlations of ALP levels with clinical parameters, including PD and GI, were reported [16,38]. These findings were similar to ours, and weak correlations were also demonstrated both in those studies and ours. With respect to CS levels, significantly higher levels were shown in destructive sites than in non-destructive or in slightly destructive sites, consistent with the findings from our previous study [14]. Interestingly, even though the ALP levels were higher in the destructive sites, no significant difference in ALP levels was found between moderate and severe destruction. On the other hand, a significant difference in CS levels between moderate and severe destruction was observed, suggesting that the CS levels can be better used than the ALP levels, to differentiate the clinical severity of periodontitis, especially between moderate and severe periodontal destruction, although the levels of both biomolecules were elevated in the GCF of patients with chronic periodontitis.

It was demonstrated in this study that either CS or ALP levels were positively correlated with all four clinical parameters, which represent periodontal destruction and inflammation. The positive correlations of elevated CS and ALP levels with increased clinical severity are in line with the findings from previous studies [14,17,35]. However, in this study, all four correlations between the clinical parameters and CS levels were stronger than those between clinical parameters and ALP levels, corresponding with the ability of CS levels, but not ALP levels, to differentiate between moderate and severe periodontal destruction as mentioned above. This may be because CS is derived only from destruction of host extracellular matrix [39], whereas ALP can be derived from both bacterial cells [40] and host cells [41-43]. Moreover, it is recognized that raised CS levels are principally due to alveolar bone resorption, whereas elevated ALP levels are found to be implicated in the process of bone formation [19] in addition to bone resorption in destructive sites [35,44]. Lastly, CS is a repeating disaccharide unit of GAGs and should not be cleaved by GCF proteinases, derived from both periodontal pathogens and host cells [45], whereas ALP enzyme can be degraded by these proteinases during GCF storage. There is still a limitation of this study due to its cross-sectional design; thus, a further longitudinal study is required to monitor any alterations in the GCF levels of these two biomolecules during periodontal disease progression in each individual tooth.

## Conclusions

In summary, CS is a better biochemical marker to differentiate moderate and severe alveolar bone destruction and shows stronger correlations with all four clinical parameters than ALP. It is, therefore, suggested by all of the findings from this study that CS is a better biochemical marker for evaluating periodontal disease severity than is ALP.

## Competing interests

All authors declare that they have no competing interests.

## Authors’ contributions

SK: Recruitment of patients and volunteers; site selection; periodontal examination; GCF sample collections. PK: Measurement of chondroitin sulfate levels. SO: Measurement of chondroitin sulfate levels. PP: Measurement of alkaline phosphatase levels. TS: Statistical analyses. DJ: Manuscript preparation. SK: Manuscript preparation and corresponding author. We would like to declare that all authors read and approved the final version of this manuscript.

## Pre-publication history

The pre-publication history for this paper can be accessed here:

http://www.biomedcentral.com/1472-6831/14/107/prepub
